# Confined Growth of NiAl-Layered Double Hydroxide Nanoparticles Within Alginate Gel: Influence on Electrochemical Properties

**DOI:** 10.3389/fchem.2020.561975

**Published:** 2020-12-02

**Authors:** Vanessa Prevot, Souad Touati, Christine Mousty

**Affiliations:** Université Clermont Auvergne, CNRS, SIGMA Clermont, ICCF, Clermont-Ferrand, France

**Keywords:** layered double hydroxides, alginate, confined coprecipitation, bionanocomposites, electrochemical behavior, permeability

## Abstract

NiAl Layered Double Hydroxide (LDH) alginate bionanocomposites were synthesized by confined coprecipitation within alginate beads. The NiAl based bionanocomposites were prepared either by impregnation by divalent and trivalent metal cations of pre-formed calcium cross-linked alginate beads or by using the metal cations (Ni^2+^, Al^3+^) as crosslinking cationic agents for the biopolymer network. The impregnation step was systematically followed by a soaking in NaOH solution to induce the LDH coprecipitation. Powder x-ray diffraction (PXRD), infrared spectroscopy (FTIR), energy dispersive X-ray analysis (EDX), thermogravimetry analysis (TGA), electron microscopies (SEM and TEM) confirmed the biotemplated coprecipitation of LDH nanoparticles ranging from 75 to 150 nm for both strategies. The drying of the LDH@alginate beads by supercritical CO_2_ drying process led to porous bionanocomposite aerogels when Ca^2+^ cross-linked alginate beads were used. Such confined preparation of NiAl LDH was extended to bionanocomposite films leading to similar results. The permeability and the electrochemical behavior of these NiAl@alginate bionanocomposites, as thin films coated on indium tin oxide (ITO) electrodes, were investigated by cyclic voltammetry, demonstrating an efficient diffusion of the K_4_Fe(CN)_6_ redox probe through the LDH@alginate based films and the improvement of the electrochemical accessibility of the Ni sites.

## Introduction

Layered double hydroxides (LDH), also termed anionic clays belong to clay minerals (Forano et al., 2013) with a brucite like layered structure and a general formula $\left[{\text{M}}_{1-\text{x}}^{2+}{\text{M}}_{\text{x}}^{3+}{\left(\text{OH}\right)}_{2}\right]{\text{A}}_{\text{x}/\text{n}}^{\text{n}-}\cdot {\text{mH}}_{2}\text{O}$, where M^2+^ and M^3+^ are divalent and trivalent cations, and A^n−^ interlayered anions. Although they are rarely observed in the natural state, this family of materials can be easily prepared in the laboratory by different methods such as coprecipitation using NaOH, urea hydrolysis, sol-gel, and epoxide methods (Tokudome et al., 2016; Prevot and Tokudome, 2017; Tichit et al., 2019). Based on fine control of the synthesis conditions, matrices with a tunable chemical composition are prepared by varying the nature of M^2+^ and M^3+^, their ratio and the type of anion intercalated which can range from simple inorganic anions, polyoxometalates (Liu et al., 2020) and organic or even bioorganic anions bearing carboxylate, sulfonate, or phosphonate groups (Taviot-Guého et al., 2018). Thanks to their unique properties, LDH are emerging as an important class of layered materials with potential applications as adsorbents in water treatment (Zhang R. et al., 2020), nuclear waste storage materials (Gu et al., 2018), electro-photo-catalysts for organic molecule conversion (Fan et al., 2014; Xu and Wei, 2018; Cai et al., 2019; Liu et al., 2020) or pollutant degradation (Zhang G. et al., 2020), energy storage and conversion (Patel et al., 2018; Cai et al., 2019; Xie et al., 2019; Yang et al., 2019), electrode materials for detection (Mousty and Prévot, 2013) and biocompatible inorganic matrices for drug delivery system development (Choi et al., 2018a,b) or biomedical imaging (Jin and Park, 2019).

Recently, the association of LDH particles with biopolymer matrices has gained increasing attention, as the formed bionanocomposites are capable, due to a synergic effect, of providing enhanced multifunctional properties for potential uses in environmental remediation and monitoring, agronomy, biocatalysis, and biomedical applications (Chatterjee et al., 2019). Special attention has been paid to LDH based bionanocomposites involving alginate, which is a large polysaccharide extracted from brown seaweeds consisting of linked α-L-guluronic acid and 1, 4 linked β-D-mannuronic acid residues (Yang et al., 2011). When intercalated within a ZnAl LDH structure, alginate was reported to provide enhanced thermal stability to the assembly and to allow the development of both potentiometric sensors and amperometric polyphenol oxidase based biosensors to determine Ca^2+^ and phenol concentration, respectively (Leroux et al., 2004; Darder et al., 2005; Sanchez-Paniagua Lopez et al., 2010). The alginate LDH assembly can also be obtained in a layer by layer approach leading to a nacre-like film with an ordered layered structure, good flexibility, and high strength (Liang et al., 2017). Interestingly, this latter approach can be applied to 3D porous support such as polyurethane foam and cotton fabrics to improve their thermal stability, flame retardancy, and smoke suppression (Liu et al., 2015; Pan et al., 2016). Another strategy to develop alginate-based bionanocomposites consisted of dispersing LDH particles in the biopolymer solution and subsequently form beads or films. Pristine LDH and calcined LDH embedded into alginate beads and magnetic alginate beads containing iron oxides displayed interesting adsorption properties toward for instance fluoride (Gao et al., 2014), phosphate (Lee and Kim, 2013; Kim, 2014), Cd^2+^, Pb^2+^, Cu^2+^ and ${\text{CrO}}_{4}^{2-}$ (Lee and Kim, 2013; Sun et al., 2018) with enhanced maximum adsorption capacity. Alginate-based drug delivery systems, developed with LDH particles intercalated with molecules such as ciprofloxacin, diclofenac, or ibuprofen (Alcantara et al., 2010; Zhang et al., 2010; Rezvani and Shahbaei, 2015), displayed an improved controlled release behavior which was explained by a limited mobility of the alginate chain due to electrostatic interactions with LDH particles, a slow-down swelling, and dissolution rates. Biohybrids based on LDH and proteins or enzymes assembly were also embedded in alginate beads to produce systems of interest for oral protein delivery and biocatalysis (Mahkam et al., 2013; Mahdi et al., 2015; Yu et al., 2019).

In parallel, an alginate assisted method was described in the literature to prepare inorganic nanoparticles, taking advantage of the alginate ability to form gels in the presence of divalent and trivalent metal cations due to their interaction with the carboxylic group on the G block of alginate forming “egg-box” like structures (Agulhon et al., 2012, 2014). Inspired by biomineralization, the confined metal cations can easily be converted either in metallic nanoparticles by a reduction step or in metal oxides nanoparticles on simple calcination under an ambient atmosphere. Pd, magnetic Co and Ni, Au metallic nanoparticles (Brayner et al., 2007; Jaouen et al., 2010; Chtchigrovsky et al., 2012), CeO_2_, NiO, TiO_2_ metal oxides, and Prussian blue type nanoparticles for instance (Primo et al., 2011; Kimling and Caruso, 2012; Tokarev et al., 2012; Wang et al., 2012), were prepared by this alginate-assisted method. The metal cations being well-distributed within the biopolymer network, it limits the crystal growth, suppresses the particle aggregation and preferentially leads to nanoparticle formation.

**Figure 1:**
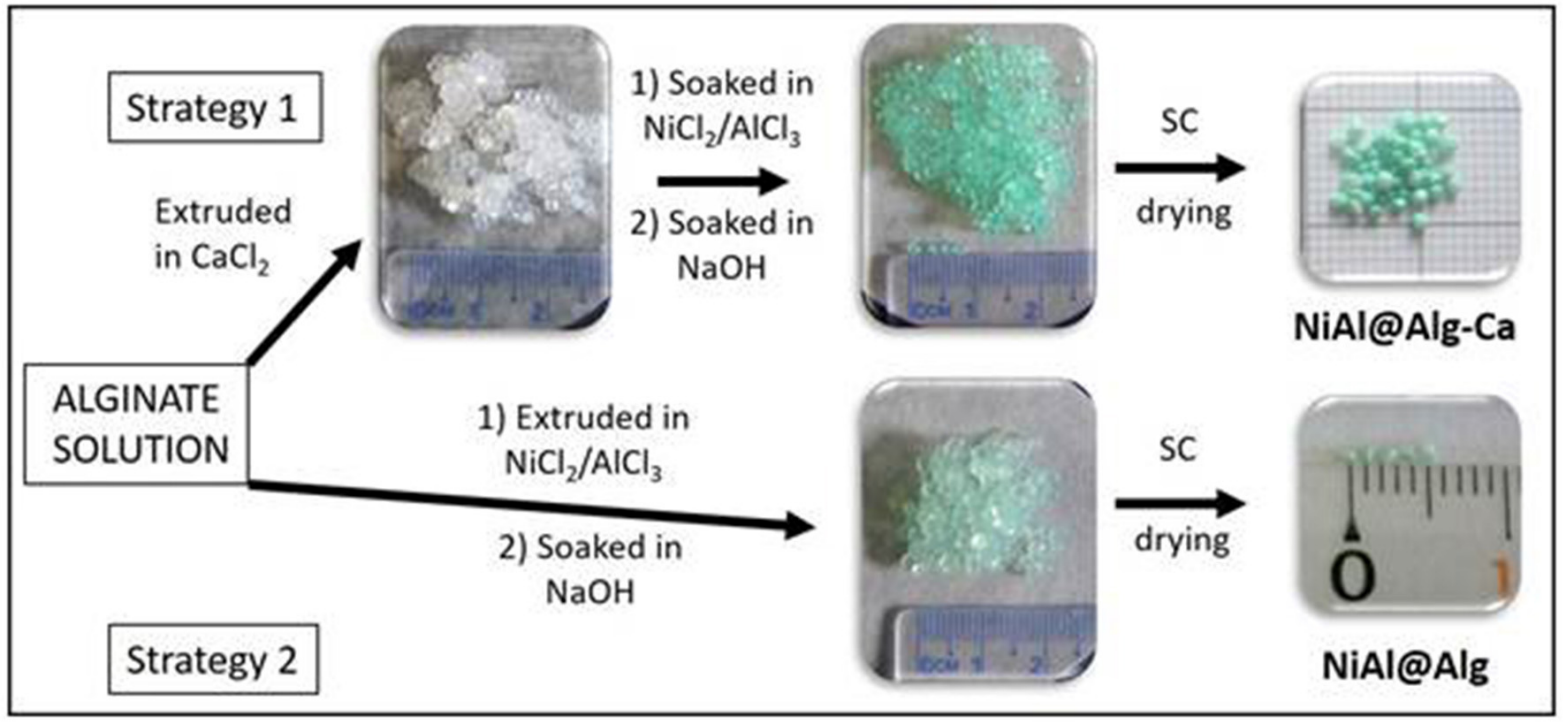
Scheme of preparation of NiAl nanoparticles confined in Alg beads using either Ca-Alg as a template for confined LDH coprecipitation (Strategy 1) or alginate cross-link by NiCl_2_ and AlCl_3_ metal salt solution (Strategy 2). For both strategies, the LDH coprecipitation was induced by soaking in a NaOH (1 M) solution and bionanocomposite beads were dried in CO_2_ supercritical conditions.

In this study, the alginate templating method was applied for the synthesis of NiAl LDH nanoparticles following two strategies (Figure 1) which involve either pre-formed calcium alginate beads or ion-exchanged nickel and aluminum alginate beads. The structures of the NiAl nanoparticles formed within the biopolymer beads, by soaking in NaOH, have been analyzed after CO_2_ supercritical drying, using PXRD, FTIR, and EDX to obtain a deep insight into the structure of the bionanocomposite aerogels. The morphology of both the aerogel beads and confined LDH nanoparticles were characterized by Scanning Electron Microscopy (SEM) and Transmission Electron Microscopy (TEM) and described in the following sections. Finally, such confined NiAl LDH coprecipitation was also extended to the preparation of bionanocomposite thin films to investigate the influence on the film permeability and the electrochemical properties of the NiAl nanoparticles formed.

## Experimental Section

### Materials and Synthesis

For all preparations, sodium alginate, NiCl_2_.6H_2_O, and AlCl_3_. 9H_2_O salts were of analytical grade. All other solvents and reagents were of commercial-grade (Aldrich, Acros Organics, Merck, Fluka). For comparison purposes, NiAl-CO_3_ and hybrid NiAl-Alg were prepared by the coprecipitation method as previously reported (Leroux et al., 2004; Faour et al., 2012). Typically, a 1 M solution of Ni and Al chloride salts (Ni^II^/Al^III^ = 2) was added under stirring and nitrogen atmosphere to a reactor containing the anion to be intercalated in a stoichiometric excess of 2 and 4 for carbonate anions and alginate, respectively. The pH of the solution was systematically maintained at 10.0 by the simultaneous addition of a 1 M NaOH solution. After addition, the suspension was allowed to stir for 24 h and then the precipitate was recovered by centrifugation, washed three times with deionized water and allowed to dry at room temperature.

For the microsphere preparation, two different strategies were followed, both using a 2 %(w/w) aqueous solution of sodium alginate. In strategy 1 (Figure 1), the polymer solution was added dropwise at room temperature to a stirred CaCl_2_ (Aldrich) solution (10 %) using a syringe with a 0.8 mm diameter needle. The formed millimeter microspheres were cured in the gelation solution for 20 min. Then, the alginate beads were soaked in a Ni and Al metal salts solution (ratio Ni^II^ /Al^III^ = 2 and [Ni^II^ + Al^III^] = 1 M) for 24 h. The alginate beads were filtrated and deeply rinsed with deionized water before to be soaked in a NaOH solution (1 M, 6 h). Finally, the beads, noted NiAl@Alg-Ca, were filtrated, washed, and keep in suspension before to be dried. In strategy 2, the biopolymer solution was directly added dropwise at room temperature to a stirred 1 M solution of Ni and Al metal salts using a syringe with a 0.8 mm diameter needle, and the beads were let in the solution for 24 h. Then as described for strategy 1, the beads, noted NiAl@Alg, were soaked in NaOH solution (1 M, 6 h), filtrated, washed, and keep in water.

The microspheres prepared by both strategies were dried by CO_2_ supercritical. Since liquid CO_2_ is not miscible with water, ethanol was used as an intermediate solvent. The microspheres were dehydrated by immersion in a series of successive ethanol-water baths at an increasing alcohol concentration (10, 20, 50, 80, 90, and 100%) for 15 min each. Finally, the microspheres were dried in an autoclave under supercritical CO_2_ conditions (105 bar, 45°C) in a Top Industry apparatus. After 6 h, the pressure was slowly decreased at constant temperature and then the autoclave was cooled down before to be opened.

### Preparation of Modified Electrodes

NiAl LDH thin films were prepared on indium tin oxide electrodes (ITO, 1 cm^2^) as conductive substrates. Before use, the ITO electrodes were cleaned by sonication (5 min) in acetone, ethanol, and deionized water, successively, and finally dried under N_2_ flow. Confined preparation of NiAl-LDH was adapted to alginate films coated on these ITO electrodes. Between each step of the preparation, the electrode was rinsed in water. The electrode was first soaked in an alginate aqueous solution (0.2 w/w) for 3 h at 60 °C, rinsed in water, and then transferred into a CaCl_2_ solution (2.75% w/w) for 15 min. This Alg-Ca/ITO modified electrode was immersed in a Ni and Al metal salt solution (1 M, Ni/Al = 2) for 24 h and then in a 0.1 M NaOH solution for 6 h. This modified electrode is referred as NiAl@Alg-Ca/ITO. The same procedure was repeated to prepare the NiAl@Alg/ITO modified electrode. In this case, the soaking step in the CaCl_2_ solution was skipped. Two other reference electrodes were prepared with 100 μL of overnight stirred 2 mg/mL NiAl-CO_3_ or NiAl-Alg suspensions deposited onto ITO electrodes and dried in air for 4 h.

### Instrumentation and Electrochemical Characterization

NiAl@Alginate bionanocomposites were characterized using different analytical techniques. X-ray diffraction patterns were recorded with a Philips X'Pert automated X-ray diffractometer using CuKα radiation (λ = 0.154051 nm), over the 2–70° (2θ). FTIR spectra were recorded with a Nicolet 5,700 spectrometer from Thermo Electron Corporation using the KBr pellet technique. Thermogravimetric analyses (TGA) were performed using a Setaram TGA92 thermogravimetric analyzer in the temperature range of 25–1,050°C, with a heating rate of 5°C min^−1^, under air atmosphere. SEM characteristics of the samples were imaged by a Zeiss supra 55 FEG-VP operating at 3 keV combined with an energy dispersive X-ray (EDX) analyzer. Specimens were mounted on conductive carbon adhesive tabs and imaged after Au sputter coating to make them conductive. N_2_ adsorption/desorption isotherms were collected in a Micromeritics ASAP2020 analyzer at −196°C. Before measurements, the samples were degassed at 80°C for 12 h. The surface area measurements were performed using the Brunauer-Emmet-Teller (BET) method. The film thicknesses were measured with an Alpha-step IQ surface profiler (KLA 134Tencor). Electrochemical measurements were made with a BioLogic Science Instruments SP-150 using a three-electrode cell, including a saturated calomel electrode (SCE) as a reference electrode, a platinum auxiliary electrode and the NiAl LDH/ITO modified electrodes as working electrodes. Cyclic voltammograms were recorded in 1 mM K_4_Fe(CN)_6_ dissolved in Tris buffer (0.1 M pH 7) and 0.1 M NaOH solution, respectively.

## Results and Discussion

### Confined NiAl LDH Coprecipitation

NiAl LDH nanoparticles were prepared via template-assisted coprecipitation within an alginate (Alg) matrix thanks to soaking in mixed metal salt solution and concentrated sodium hydroxide solution (Figure 1). Such confined LDH coprecipitation by successive impregnations was previously described by our group to successfully lead to three-dimensional macroporous LDH using polystyrene colloidal crystal as a sacrificial hard template (Géraud et al., 2008). In a first strategy, Ca-alginate beads, obtained by alginate ion cross-link in a calcium chloride solution, were submitted to a solvent exchange process to introduce Ni^2+^ and Al^3+^ in the hydrogel network. The infiltration of the metal cation solution was traduced by the color change of the beads from white to light green (Figure 1), demonstrating the efficient diffusion of the solution within the biopolymer network. Since the pH of the infiltrated metal salt solution is ~3.0, Al^3+^ and Ni^2+^ are the main infiltrated species, although the presence of Al(OH)^2+^ and $\text{Al}{\left(\text{OH}\right)}_{2}^{+}$ cannot be excluded. No modification of the bead size and aspect was observed during infiltration and subsequent soaking in sodium hydroxide solution. To be able to characterize the Alg-Ca bionanocomposites by solid-state techniques, the beads were dried. While drying in a stove at 40°C let to a net diminution of the bead size due to an important shrinkage of the alginate network, the drying using supercritical CO_2_ (CO_2_ SC) conditions allowed to preserve the sphericity of the beads and limit the size decrease. TGA analyses (Supplementary Figure 1) of the sodium alginate used as a precursor and the Alg-Ca beads dried by the two different ways revealed that systematically the thermal decomposition occurred in four steps around 120°C, 200–240°C, 510–600°C and 675–1,010°C, leading to a total mass loss comprised between 93.5 and 88.1% (Table 1 and Supplementary Table 1). The decomposition steps can be attributed according to the literature (dos Santos Araújo et al., 2019) to the dehydration of the matrix, the biopolymer decomposition, the corresponding carbonate salt formation (Na_2_CO_3_ and CaCO_3_) and at higher temperature their decomposition. Note that the thermal profiles below 200°C, corresponding to the dehydration step, showed a net difference in the water amount into the beads with 14.7 and 44.3% of mass loss for the Alg-Ca beads dried in the stove and CO_2_ SC conditions, respectively. This can be attributed to the ability of CO_2_ SC conditions to better maintain the hydration rate into the aerogel network. Since the dehydration step occurred at a lower temperature, the CO_2_ SC conditions seem also to favor the diffusion and the mass transfer in the biopolymer network.

**Table 1:** Main structural characteristics of the LDH and alginate-based bionanocomposites.

**Sample**	**d_**inter**_** **(nm)**	**a** **(nm)**	${\text{D}}_{006}\text{/}{\text{D}}_{110}^{*}$ (nm)	**Ni/Al** **ratio**	**BET** **(m^**2**^/g)**	**Mass loss** **(%)**
Alg-Ca	–	–	–	–	168	91
NiAl@Alg-Ca	0.75	0.304	1.98/3.96	3.5	423	49
NiAl@Alg	0.74	0.301	2.08/3.50	3.2	33	51
NiAl-Alg	1.27	0.299	–	–	–	55
NiAl-CO_3_	0.76	0.299	2.68/3.11	2.1	72	39

*determined using Scherrer equation.

**Figure 2:**
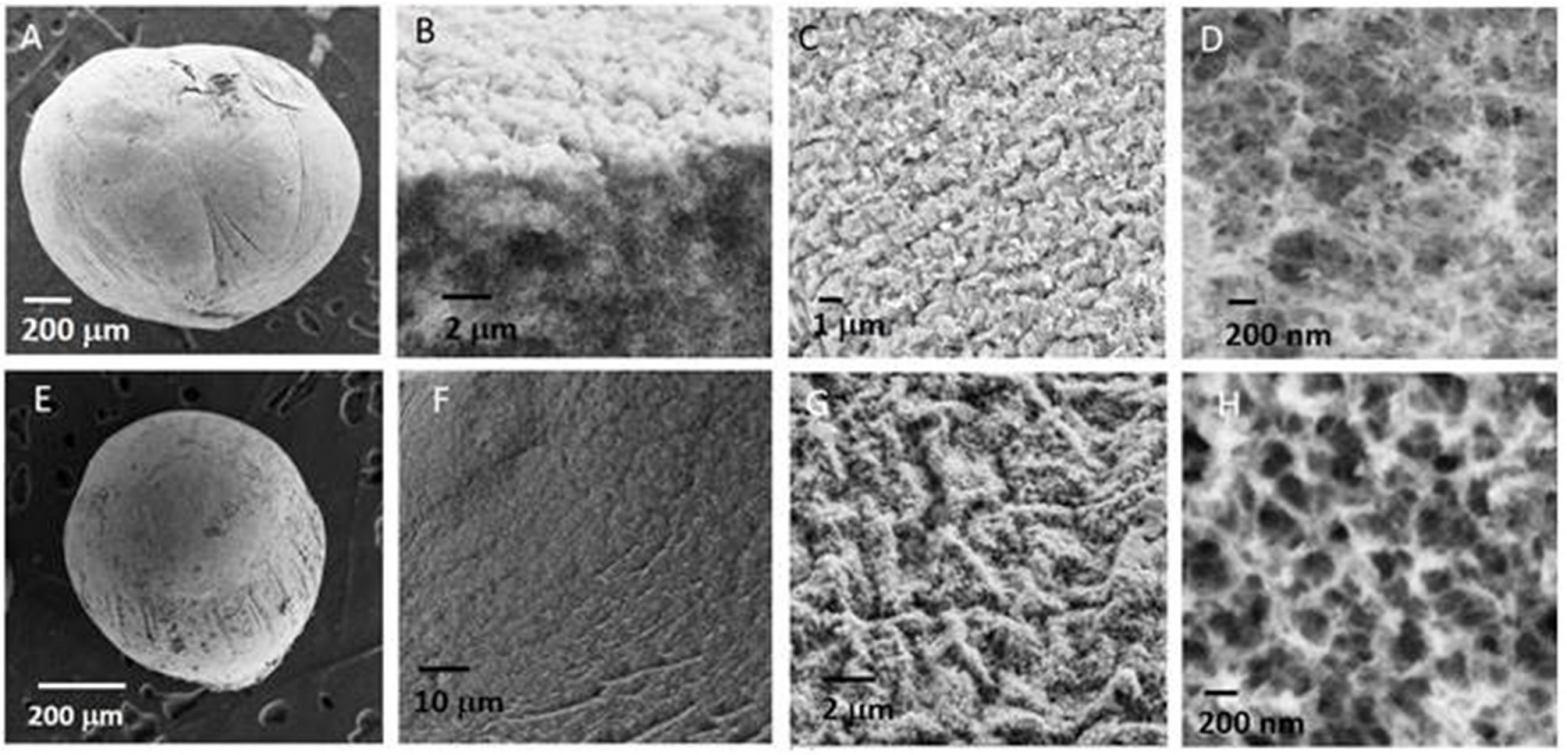
SEM images of **(A–D)** NiAl@Alg-Ca beads and **(E–H)** NiAl@Alg beads dried upon CO_2_ supercritical conditions.

Then, in the following the biopolymer beads were systematically dried using this latter technique leading to beads with the size of the order of a millimeter. SEM images of a NiAl@Alg-Ca aerogel (Figures 2A–D) showed their morphology was similar to the one observed for Alg-Ca precursor beads (Supplementary Figure 2) with a well-defined smooth surface, while images at higher magnification revealed a nested network based on small filaments, characteristic of biopolymer hydrogel. The presence of NiAl particles within the network was very difficult to be distinguished. In a second strategy, the biopolymer network was directly cross-linked by the addition of the sodium alginate solution in the Ni^2+^ and Al^3+^ chloride solution.

**Figure 3:**
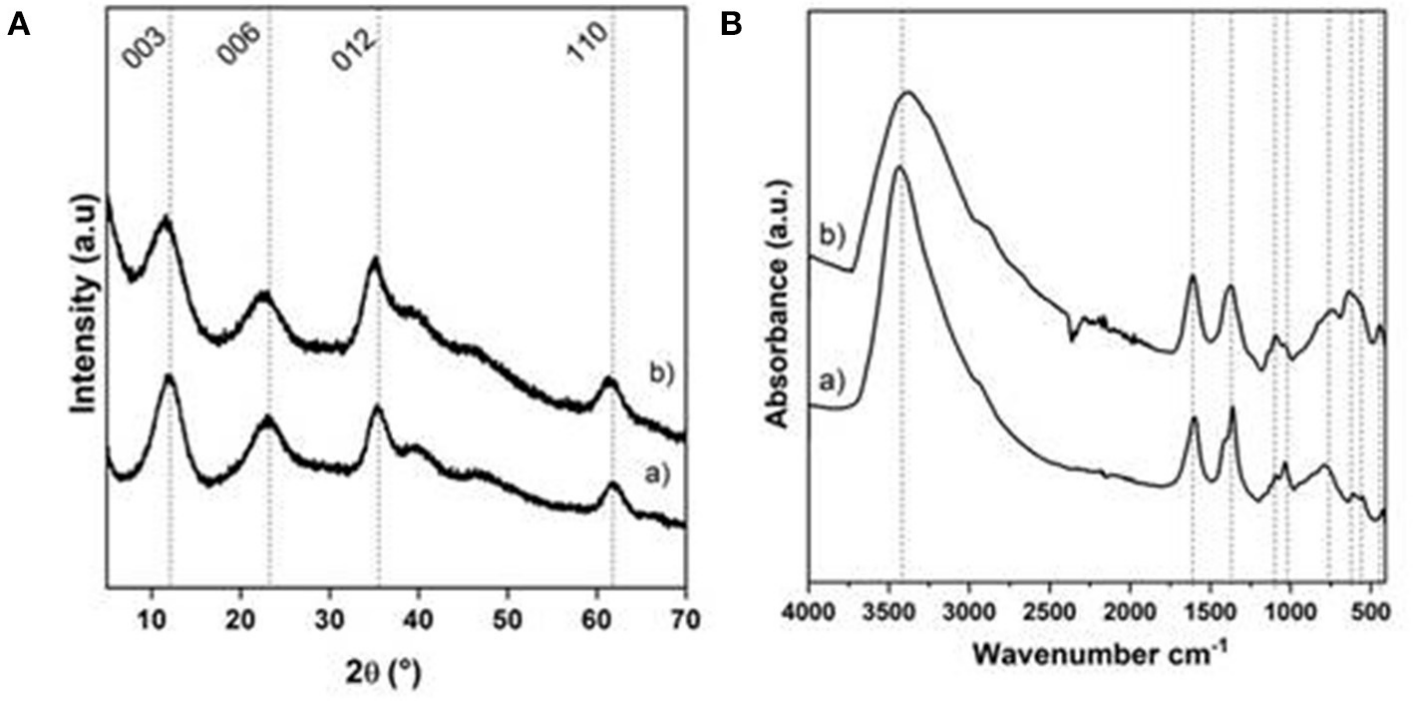
**(A)** PXRD patterns and **(B)** FTIR spectra of (a) NiAl@Alg-Ca beads and (b) NiAl@Alg beads dried upon CO_2_ supercritical conditions.

It is known that not only Ca^2+^ displays the ability to interact with the G blocks of the alginate to form an egg-box structure and ionotropic gelation, other divalent and trivalent cations, such as Cu^2+^, Ba^2+^, Co^2+^, Ni^2+^, and Zn^2+^, can also be used (Agulhon et al., 2012, 2014), even if their affinity toward alginate can differ. Following this approach and subsequent soaking in NaOH, NiAl@Alg beads were formed with a size slightly higher compared to the NiAl@Alg-Ca, which can be attributed to a lower affinity of the cations Ni^2+^ and Al^3+^ for alginate compared to Ca^2+^, leading to a less reticulated and dense gel. Such properties of the beads induced after the CO_2_ SC drying, a more pronounced shrinkage of the NiAl@Alg aerogel beads displaying an average size below one millimeter. As previously described for NiAl@Alg-Ca, the SEM images showed the morphology of the beads with a smooth surface and in the inner a network characteristic of a biopolymer (Figures 2E–H). To get further insight on the NiAl LDH coprecipitation within the two kinds of alginate beads (Strategy 1 and 2), PXRD and FTIR analyses were carried out (Figure 3).

Compared to the amorphous structure of the Alg-Ca beads (Supplementary Figure 3A), the PXRD patterns of the NiAl@Alg-Ca and NiAl@Alg beads (Figure 3A) displayed the characteristic diffraction lines of hexagonal LDH structure, which crystallizes in an R-3m space group, with especially the 003 and 006 reflections, traducing the stacking in the layered structure and the 110 reflections related to the octahedral sheets and the interatomic distance. Such diagrams for the beads revealed as expected, the NiAl LDH formation within the biopolymer network for both strategies. In all the case, the width of the diffraction lines appeared wide reflecting small coherent domains for both D_006_ and D_110_ (Table 1) and a low level of crystallinity comparable to the coprecipitated NiAl-CO_3_ phase.

The interlayer distance observed (Table 1) is in good agreement with the presence of carbonate anions in between the LDH layer since carbonate anions displayed a high affinity toward LDH matrices and no precaution was taken during the coprecipitation.

Noticeably, the conditions used in this work were not in favor of alginate intercalation described when LDH was coprecipitated in a sodium alginate solution (Leroux et al., 2004), which lead to an interlayer domain expansion and an interlamellar distance of 1.27 nm (Supplementary Figure 4). The anionic carboxylic groups being already involved in the egg box alginate structure; we can hypothesize that they were no more available to compensate the positive charge of the NiAl LDH layer during the coprecipitation. Further evidence of the presence of carbonate anions was provided by FTIR spectroscopy (Figure 3B) due to the presence of a band at 1,371 cm^−1^ corresponding to the stretching band of the carbonate anions. In the same area, the ν_as_ and ν_s_ of the carboxylate group of the alginate can also be observed at 1,589 and 1,417 cm^−1^. Note that in the case of NiAl@Alg beads, even if it was difficult to distinguish between the two bands ν_s__COO−_ and the ν_CO3_ which appeared as one single large band, the change in ν_as_/ν_s_ intensity ratio compared to sodium alginate reference spectrum (Supplementary Figure 5B) is in favor of the carbonate presence. It is noteworthy that in addition to the alginate and carbonate bands, the LDH lattice vibrations (ν_M−O_ and ν_O−M−O_) were observed on the spectra below 900 cm^−1^.

**Figure 4:**
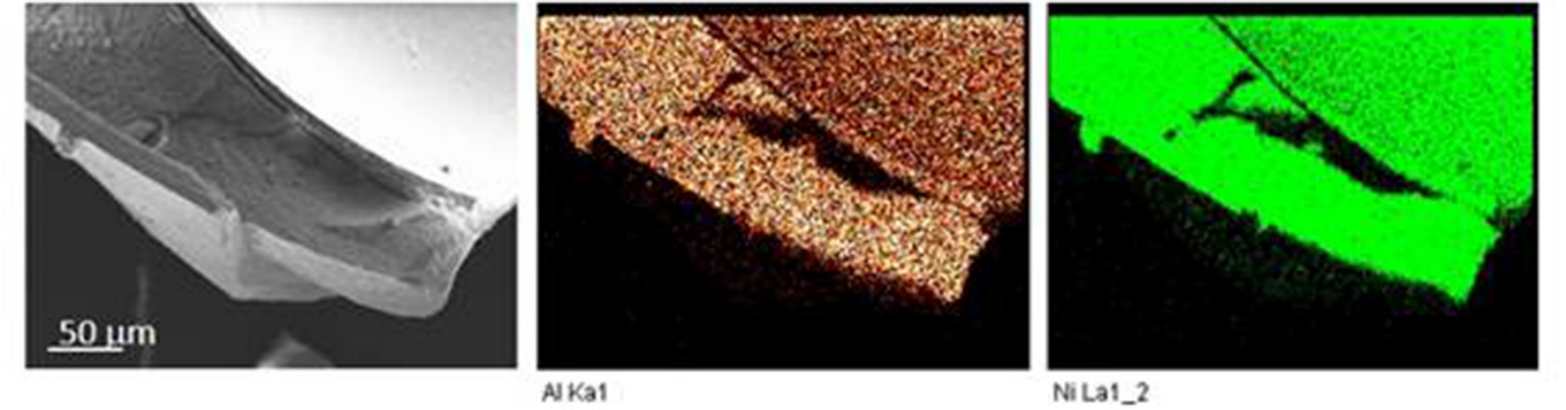
Energy dispersive X-ray (EDX) mapping analysis of NiAl@Alg-Ca.

The chemical composition and particularly the Ni/Al ratios of the different beads were obtained by EDX (Supplementary Figure 6). The slight difference for the Ni^2+^/Al^3+^ ratio was observed compared to the ratio of 2 used in the precursor solution. Systematically higher ratios (Table 1) were measured which may be due to either a preferential infiltration and affinity of Ni^2+^ within the polymer network compared to Al^3+^ or the high pH used for the LDH formation during the soaking process in concentrated 1 M NaOH, which could favor the formation of $\text{Al}{\left(\text{OH}\right)}_{4}^{-}$ species in solution rather than its precipitation within the LDH layers. Interestingly, EDX mapping showed a homogeneous repartition of the Ni^2+^ and Al^3+^ elements within the beads (Figure 4), traducing that the alginate template-assisted coprecipitation induced a good repartition of the LDH particles within the biopolymer network without segregation.

**Figure 5:**
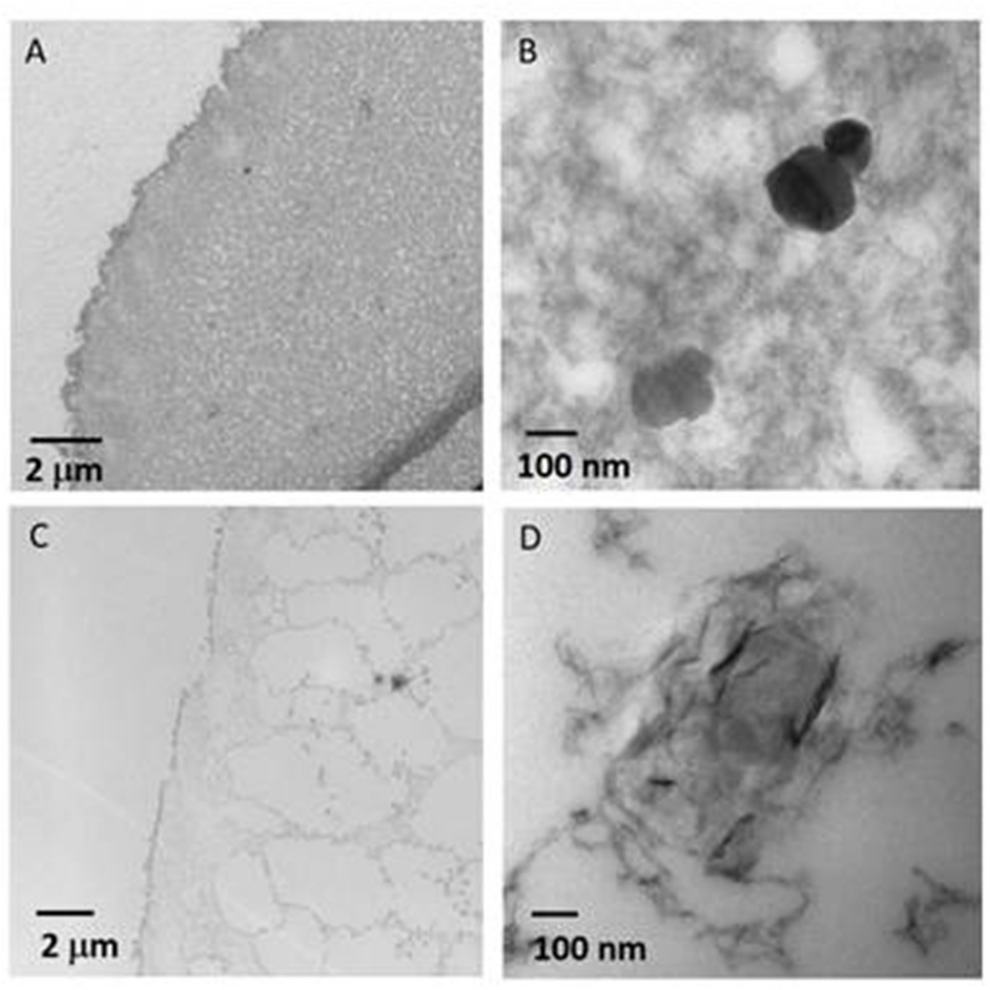
TEM images of ultrathin sections obtained by ultramicrotomy from wet **(A,B)** NiAl@Alg-Ca beads, and **(C,D)** NiAl@Alg beads.

The observation by TEM of ultra-thin section obtained from the wet NiAl@Alg-Ca beads included in a resin (Figures 5A,B) showed the presence of a dense biopolymer network, in good agreement with the SEM images. At higher magnification, dispersed well-defined hexagonal LDH platelets were observed which seemed to be embedded within the biopolymer. These observations strongly contrast with the images obtained from NiAl@Alg beads (Figures 5 C,D) which indicated a much-opened network for alginate which may be correlated to the lower affinity of alginate for Ni^2+^/Al^3+^ compared to Ca^2+^ as previously discussed. Moreover, a completely different LDH particle shape was observed for the NiAl@Alg beads. The NiAl- nanoparticles strongly interacted with the biopolymer chains and were mainly observed on the edge. Such morphology is to be related to the use of Ni^2+^ and Al^3+^ as cross-linking ions in the second strategy which imposes a strong interaction between the biopolymer molecular chain and the metal cations to be involved in the LDH layers.

**Figure 6:**
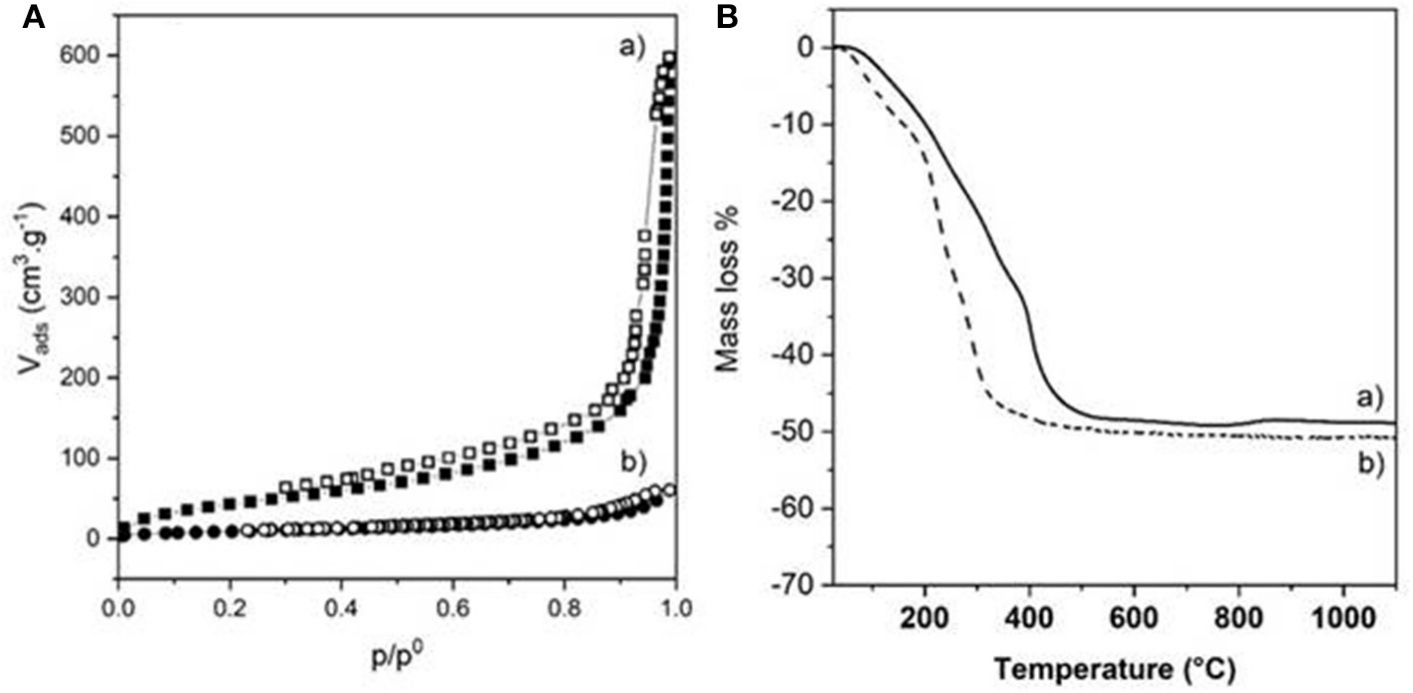
**(A)** N_2_ adsorption-desorption isotherms and **(B)** TGA curves of (a) NiAl@Alg-Ca beads and (b) NiAl@Alg beads dried upon CO_2_ supercritical conditions.

The choice of the strategy followed also impacted strongly the textural properties of the dried bionanocomposite beads, as highlighted by the N_2_ adsorption-desorption isotherms (Figure 6A). The two isotherms displayed the same characteristic shape (Type IV with hysteresis loop is H3) of a mesoporous material, also observed for Alg-Ca beads (Supplementary Figure 3). However, successive infiltrations of pre-formed Alg-Ca beads (Strategy 1) led to more porous NiAl@Alg-Ca beads with a high BET surface area of 423 m^2^ g^−1^ with a pore volume of 0.81 cm^3^/g. The pore size distribution showed well-defined mesopores with a size centered at 3.5 nm mainly distributing from 2.0 to 5.0 nm (Supplementary Figure 7). We assumed that the shrinkage observed during drying for strategy 2 strongly reduced the beads' porosity (Table 1).

**Figure 7:**
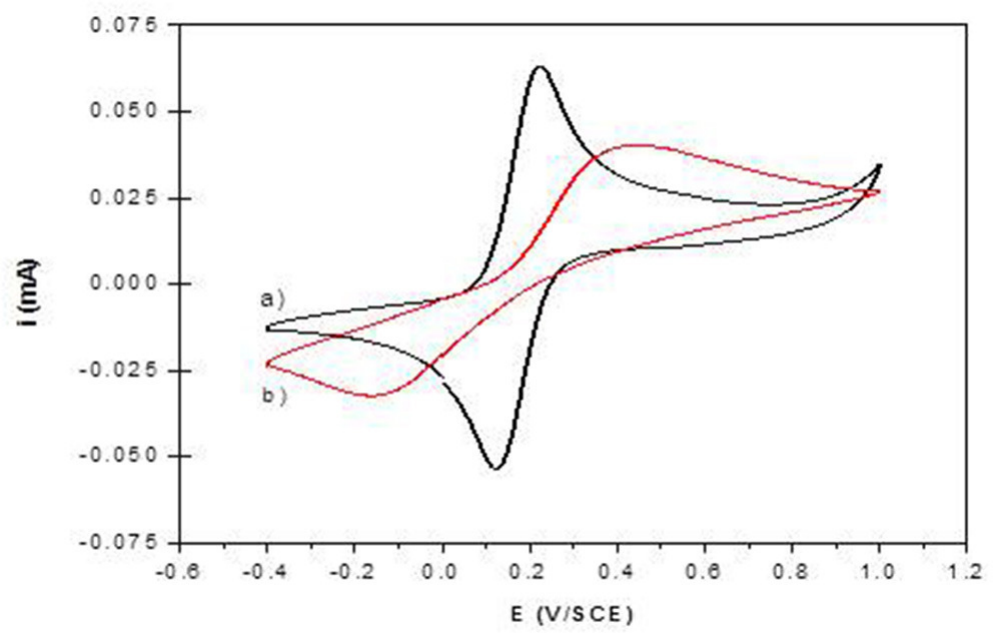
Cyclic voltammograms of 1 mM K_4_Fe(CN)_6_ in 0.1 M Tris buffer (pH 7) recorded at (a) NiAl@Alg-Ca/ITO and (b) NiAl-CO_3_/ITO (v = 50 mVs-1).

In parallel, the presence of the LDH nanoparticles within the biopolymer networks had an important influence on the thermal behavior of the materials (Figure 6B and Table 1). On one hand, the decomposition process of the NiAl@Alg aerogel beads occurred at a lower temperature compared to NiAl@Alg-Ca aerogel, which may be explained by biopolymer combustion favored by the proximity with the NiAl LDH nanoparticles and the derived NiAl LDH mixed oxides formed at the intermediate temperatures. However, systematically the presence of NiAl LDH nanoparticles delayed the decomposition in the low-temperature range (Supplementary Figure 1). On the other hand, for both types of bionanocomposite beads, the TGA curves showed a complete decomposition at 500°C with a fairly similar total mass loss close to 50% and very different thermal profiles from that observed for pure Alg-Ca beads (Supplementary Table 1 and Supplementary Figure 1). The decomposition steps of the biopolymer and the inorganic matrix (dehydration/dehydroxylation/decarbonation) are overlapped preventing a clear description of the decomposition. The net decrease of the total mass loss compared to Alg-Ca beads (Table 1) allowed to estimate the amount of NiAl within the bionanocomposites at ~80% in mass. These results highlighted that the alginate hydrogels were efficient to confine the coprecipitation of a large amount of NiAl nanoparticles.

### Toward 2-D NiAl@Alginate Modified Electrodes

The relevance of Ni-based LDH in electrochemical applications, such as electrochemical analysis (Tonelli et al., 2013), energy storage (Patel et al., 2018), electrocatalytic oxygen evolution (OER) (Anantharaj et al., 2017) or electrochromism (Mondal and Villemure, 2012; Martin et al., 2016), has been widely described in the literature. NiAl LDH modified electrodes were prepared by different procedures, for instance by the solvent casting of aqueous suspensions of LDH prepared by the coprecipitation (Vialat et al., 2013; Taviot-Gueho et al., 2016) or the polyol method (Faour et al., 2012) and by the direct electrodeposition on the electrode surface (Scavetta et al., 2007; Tonelli et al., 2013; Mousty and Walcarius, 2015). All these cited works describe the importance of the particle size, the aggregation state of LDH particles, and the presence of structural defects on the electrochemical activity of nickel sites. The confined coprecipitation of NiAl LDH described previously was therefore extended to alginate films coated on ITO electrodes to characterize the electrochemical accessibility of Ni sites in these bionanocomposite materials.

Thin films of alginate, reticulated or not with Ca^2+^, were prepared on ITO as described in the literature (Yang et al., 2010) (see the experimental part). An ITO electrode was chosen to improve the adhesion of the biopolymer in comparison to a Pt surface. These homogeneous films, as shown in SEM images (Supplementary Figure 8), have a thickness of 1.1 and 0.62 μm, respectively, before and after the reticulation step. The NiAl@Alg-Ca/ITO and NiAl@Alg/ITO electrodes were prepared by impregnation of these as-prepared alginate films by a Ni and Al metal salt solution, as previously described for the alginate beads. The formation of the confined NiAl LDH phase was confirmed by PXRD leading to a similar diagram to the one described previously (Supplementary Figure 8 and Figure 3). The formation of the NiAl LDH phase within the alginate films caused a decrease of the film thickness (Table 2) and some cracks appear in the films (Supplementary Figure 8). For comparison, two other electrodes, namely NiAl-Alg/ITO and NiAl-CO_3_/ITO, were prepared by the deposition of 200 μg/cm^2^ of LDH suspensions. The thicknesses of these films were slightly higher (Table 2).

**Table 2:** Characterization of the NiAl LDH/ITO modified electrodes.

**LDH/ITO**	**Thickness** **(μm)**	${\text{D}}_{\text{app}}^{\text{a}}$ **(cm^**2**^ s^**−1**^)**	${\text{Ip}}_{\text{a}}^{\text{b}}$ **(mA cm^**−2**^)**
NiAl@Alg-Ca	0.41	7 × 10^−7^	5.3
NiAl@Alg	0.40	5 × 10^−7^	1.6
NiAl-Alg	0.91	2 × 10^−7^	0.08
NiAl-CO_3_	2.00	9 × 10^−8^	0.04

^a^*Apparent diffusion coefficient calculated for 1 mM K_4_Fe(CN)_6_*, ^b^*Anodic peak current recorded in 0.1 M NaOH at v = 50 mVs^−1^*.

**Figure 8:**
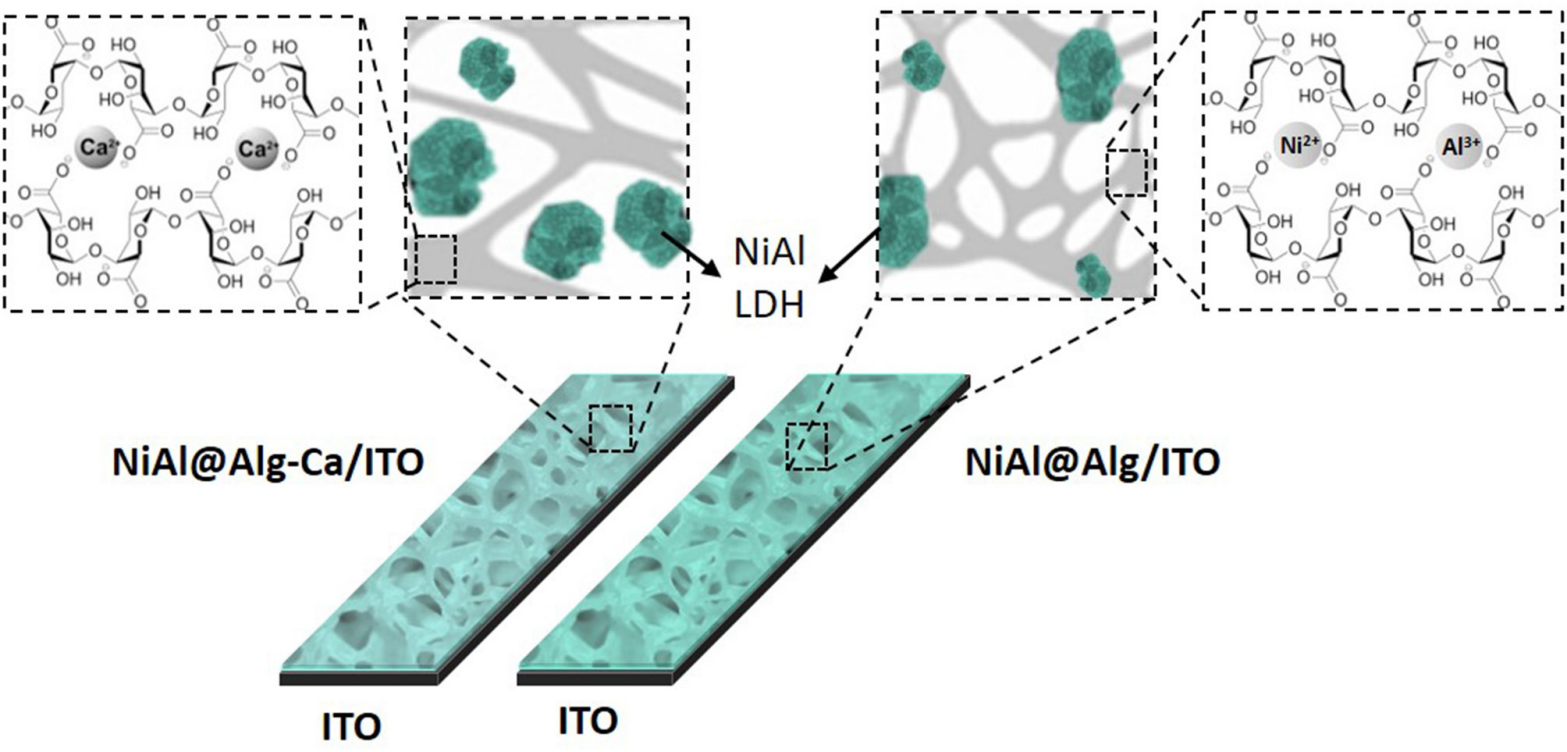
Schematic representation of the NiAl@Alg-Ca/ITO and NiAl-Alg/ITO.

The permeability of the NiAl@Alg and NiAl@Alg-Ca thin films was investigated with potassium ferrocyanide as an electroactive probe, showing a reversible signal in cyclic voltammetry (CV) (Supplementary Figure 9). Based on the variation of the anodic current (ip_a_/A) as a function of square root of the scan rate (*v*^1/2^), apparent diffusion coefficients (D_app_) were calculated from Randles-Sevcik equation: ip = 2.69 × 10^5^ n^3/2^ A.C v^1/2^ D^1/2^, where *n* = 1e^−^ transfer, C = 1 × 10^−6^ mol cm^3^, A = electrode surface (1 cm^2^). The calculated values of D_app_ are compared to those obtained with the NiAl-Alg and NiAl-CO_3_ modified electrodes (Supplementary Figure 9 and Table 2). The diffusion of the electroactive probe through the NiAl@Alg composite films was not perturbed, with a linear variation of the Ip_a_ fct √*v* over the whole investigated scan rate range (Figure 8 and Supplementary Figure 9). However, with the NiAl-CO_3_/ITO, the electron transfer became slower with an increase of ΔEp and smaller peak intensities. In this case, the origin of the straight line differed from zero and the calculated D_app_ value was very small, suggesting the possible accumulation of the redox probe $\text{Fe}{\left(\text{CN}\right)}_{6}^{4-}$ and the hindrance of its diffusion within the film. It should be noted that the film was thicker in this latter case. With the NiAl-Alg, the situation seems to be intermediate with a possible accumulation of the probe in the film but with higher mobility. We assumed that the confined coprecipitation of the NiAl LDH nanoparticles within the porous network of the biopolymer favor the diffusion and the film permeability as illustrated in Figure 8.

**Figure 9:**
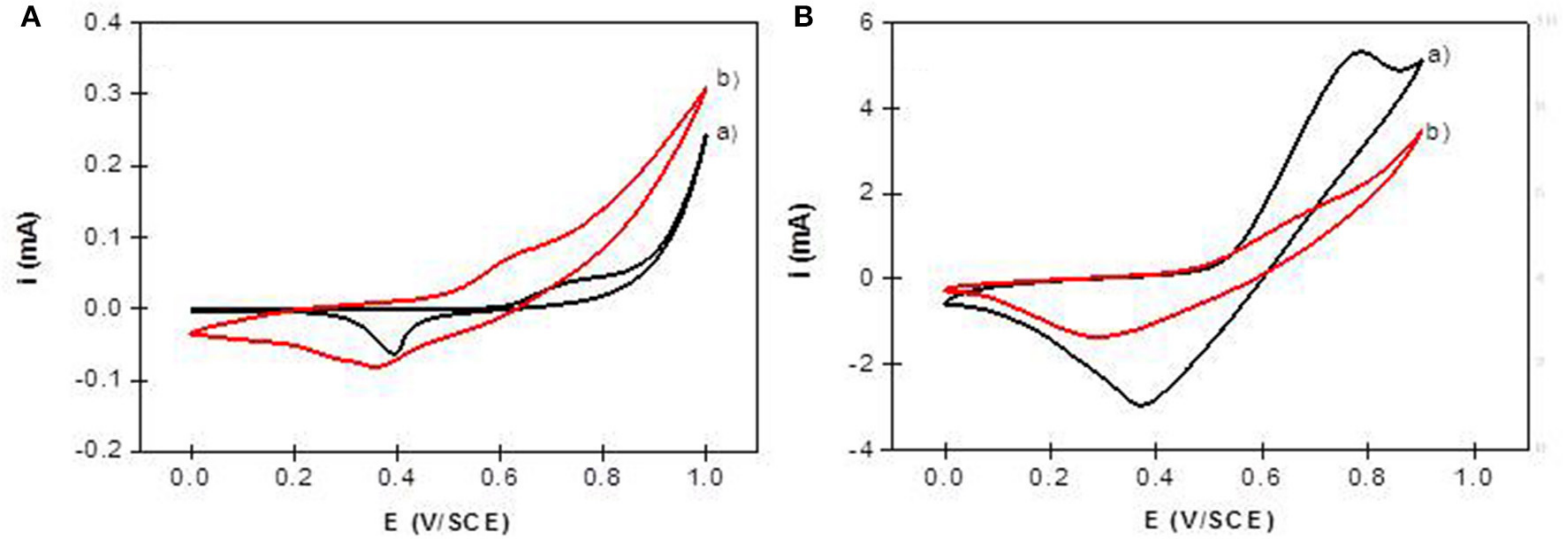
Cyclic voltammograms recorded in 0.1 M NaOH (*v* = 50 mVs^−1^) using **(A)** NiAl-CO_3_/ITO (curve a) and NiAl-Alg/ITO (curve b) modified electrodes prepared by solvent casting; **(B)** NiAl@alginate composite electrodes prepared by impregnation (a) NiAl@Alg-Ca/ITO and (b) NiAl@Alg/ITO.

The electrochemical behavior of NiAl LDH in an alkaline medium is characterized in cyclic voltammetry by a quasi-reversible one-electron transfer Ni(II)/Ni(III), defined by an anodic peak and its corresponding cathodic peak situated between 0.40 and 0.80 V/SCE. In a previous study, we have shown that the peak position and intensity depend on the pH of the electrolyte solution and the nature of the alkali cations; the best signal being obtained in NaOH (Vialat et al., 2013; Taviot-Gueho et al., 2016). NiAl-CO_3_/ITO displayed a typical CV signal corresponding to the oxidation of the Ni sites in the LDH layer (Figure 9A). It should be noted that the electron transfer seems to be slightly slowdown at the ITO interface, with a higher ΔEp compared to that generally observed at a Pt electrode (Vialat et al., 2015). With NiAl-Alg/ITO, the electrochemical signal seems to be slightly enhanced, probably due to the better permeability of the film which allows better diffusion of the electrolyte ions. Interestingly, the NiAl@alginate bionanocomposites displayed more intense signals (Figure 9B), with a particularly high anodic current for the NiAl@Alg-Ca/ITO (Table 2). Indeed, this current density, recorded at 50 mVs^−1^ in 0.1 M NaOH (5.3 mA cm^−2^), is two times larger than the value (2.5 mA cm^−2^) we previously found with a Pt electrode modified with a NiAl-CO_3_ compound, prepared by a glycine-assisted hydrothermal method, where the presence of 2H1 stacking motifs in the 3R1 LDH lattice results in an enhanced electrochemical signal (Faour et al., 2012). This shows that Ni sites in LDH nanoparticles obtained by template-assisted coprecipitation in the alginate matrix are more accessible for an electron transfer.

## Conclusion

In this paper, we reported that the bioinspired alginate-assisted coprecipitation of NiAl LDH nanoparticles was possible using two different strategies. The first one based on successive impregnations of alginate beads formed by extrusion in a CaCl_2_ solution allowed the formation of well-defined carbonate intercalated NiAl nanoparticles distributed within the biopolymer network. The beads dried in CO_2_ supercritical conditions displayed high surface area and were stabilized in the low-temperature range. The second strategy involved ion cross-linking of the alginate directly by the metal cations expected to coprecipitate and form the LDH layers. In this case, the hydrogel was less reticulated and the formed NiAl nanoparticles strongly interacted with the biopolymer chains. For both strategies, the bionanocomposite aerogels contained ~80% by mass of LDH, which demonstrated the efficiency of the coprecipitation process carried out by successive impregnations. Interestingly, we showed these confined approaches can be extended to the preparation of thin bionanocomposite films which were evaluated as modified electrodes. Our results indicated that a better diffusion was achieved for the NiAl nanoparticles formed into the hydrogel compared for instance to the NiAl phases intercalated by anionic alginate. Moreover, the synthetic conditions used enhanced the accessibility of the Ni sites within the LDH layers. These template-assisted strategies can be considered as a simple, low cost and environmentally-friendly route to prepare LDH which can be extended to other hydrogels and LDH chemical compositions opening the way to new LDH based bionanocomposite materials.

## Data Availability Statement

The raw data supporting the conclusions of this article will be made available by the authors, without undue reservation.

## Author Contributions

VP and CM (CNRS senior researchers) were in charge of designing the experiments, writing, and revising the manuscript. ST (Ph.D. student) performed the experiments and the electrochemical measurements. All authors contributed to the article and approved the submitted version.

## Conflict of Interest

The authors declare that the research was conducted in the absence of any commercial or financial relationships that could be construed as a potential conflict of interest.
