# Supplementary material for: Confined Growth of NiAl-Layered Double Hydroxide Nanoparticles Within Alginate Gel: Influence on Electrochemical Properties
Source: Front Chem. 2020 Dec 2;8:561975. doi: 10.3389/fchem.2020.561975 (PMC7738438; doi:10.3389/fchem.2020.561975)
Supplement: Supplementary file 1 [file Data_Sheet_1.pdf]

Supplementary Material

Confined Growth of NiAl Layered Double Hydroxide Nanoparticles Within Alginate Gel: Influence On Electrochemical Properties

Vanessa Prevot*, Souad Touati, Christine Mousty*

Université Clermont Auvergne, CNRS, SIGMA Clermont, ICCF, F-63000 Clermont-Ferrand, France

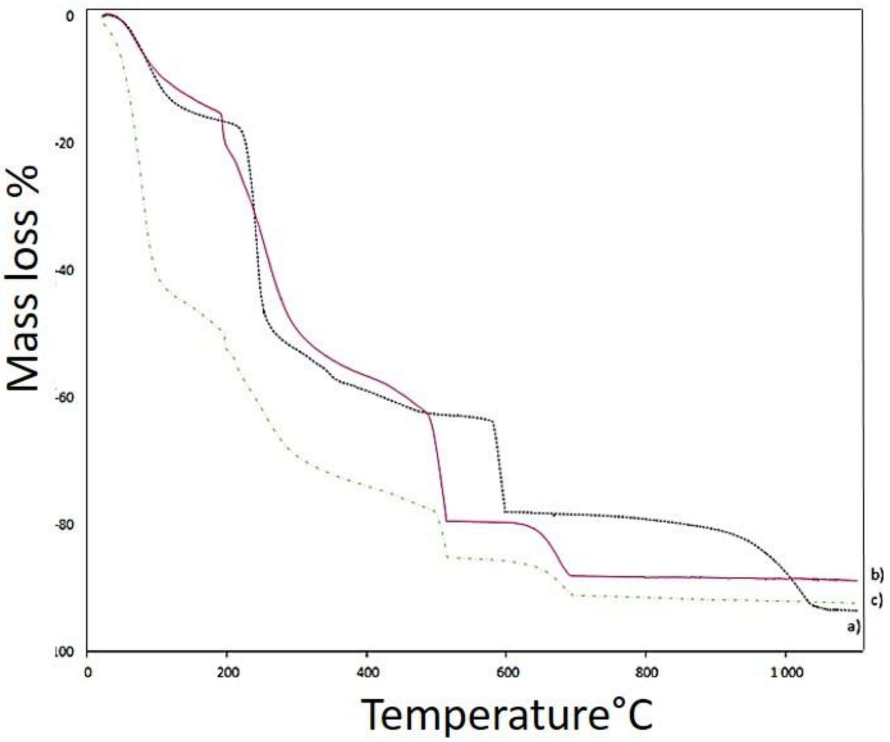

Figure S1: TGA curves of a) Na-Alginate, b) Alg-Ca beads dried in the stove at 40°C and C) CO_2 SC dried Alg-Ca beads

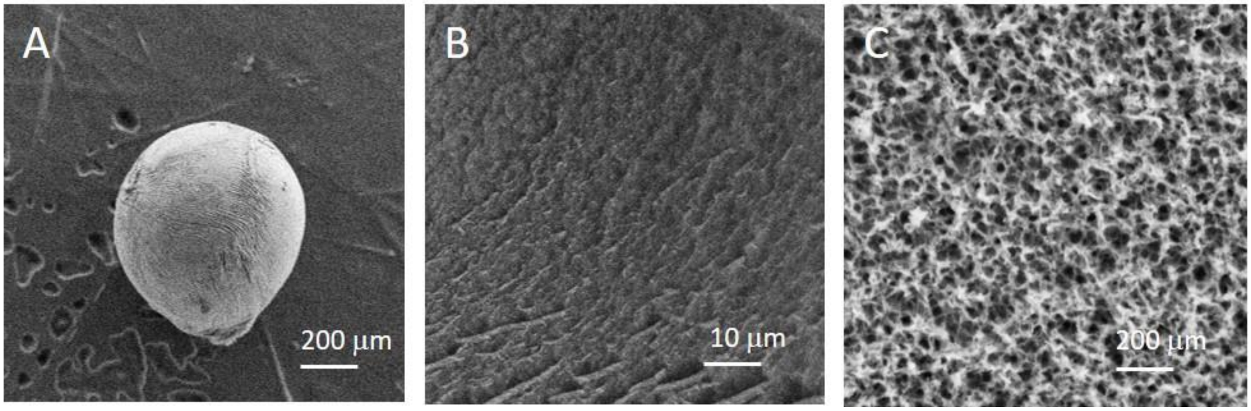

Figure S2: SEM images of a Alg-Ca bead at different magnifications

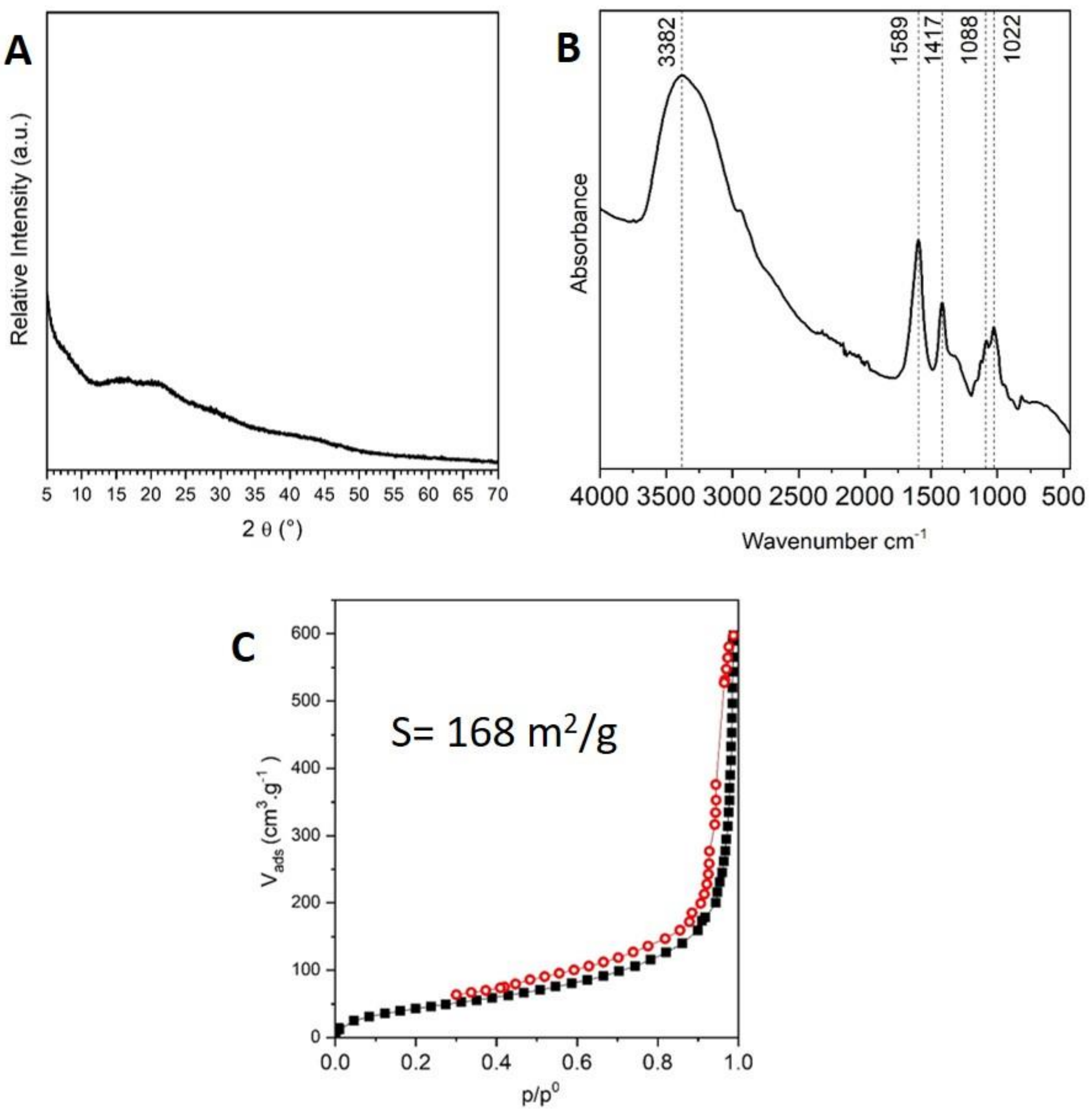

Figiure S3: PXRD pattern, FTIR spectrum and N_2 adsorption-desorption of Alg-Ca beads at different magnifications

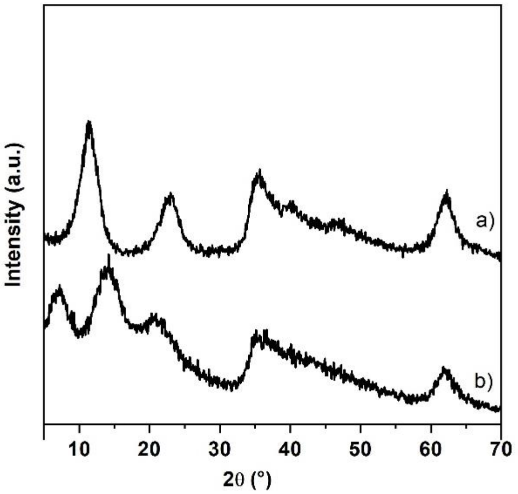

Figure S4: PXRD patterns of NiAl-CO_3 LDH phase coprecipitated a constant pH, b) hybrid NiAl-Alginate coprecipitated in presence of an alginate solution.

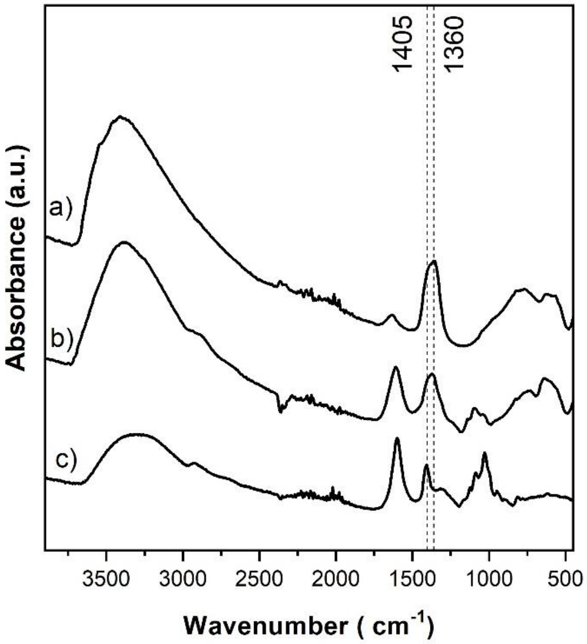

Figure S5: FTIR spectra of a) NiAl-CO_3 LDH phase coprecipitated a constant pH, b) NiAl@Alg-Ca beads and c) sodium alginate

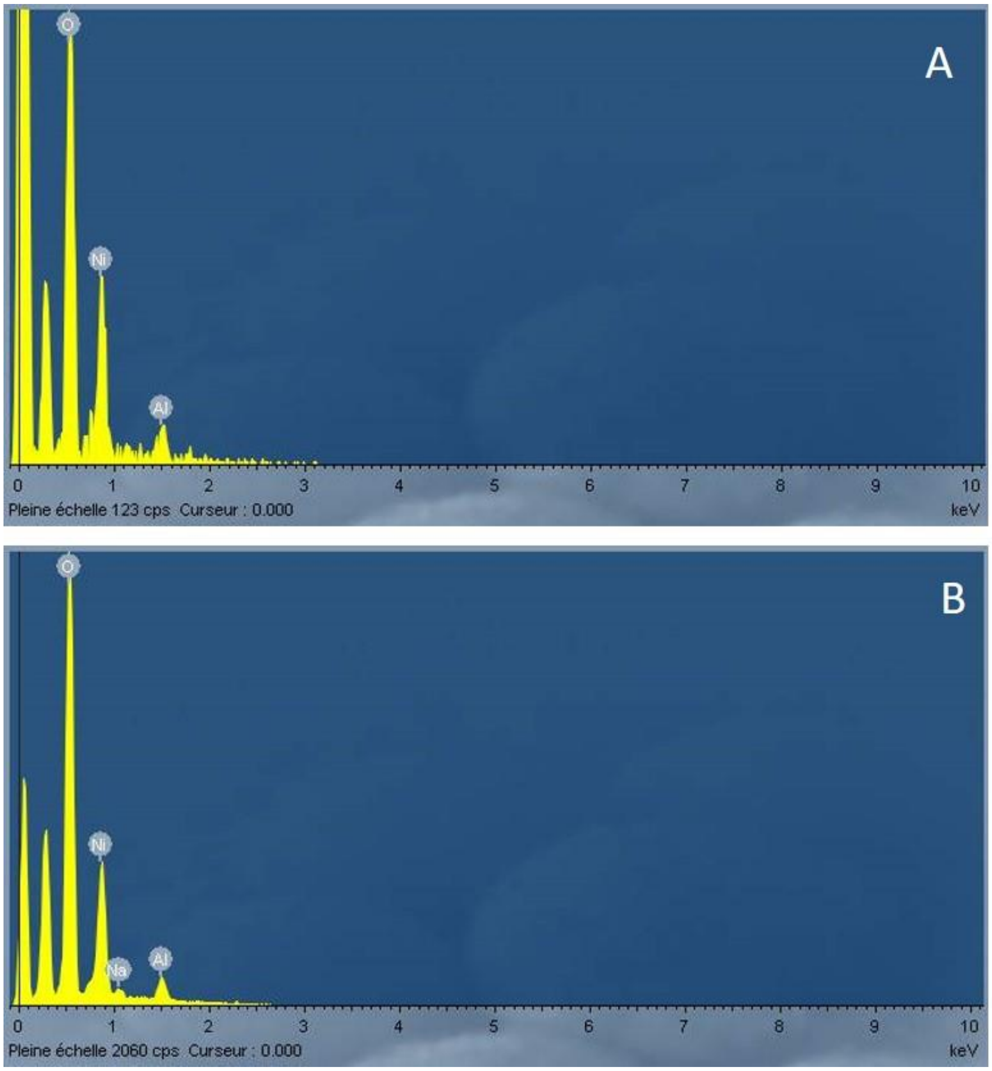

Figure S6: EDX analysis of A) NiAl@Alg-Ca beads and A) NiAl@Alg beads

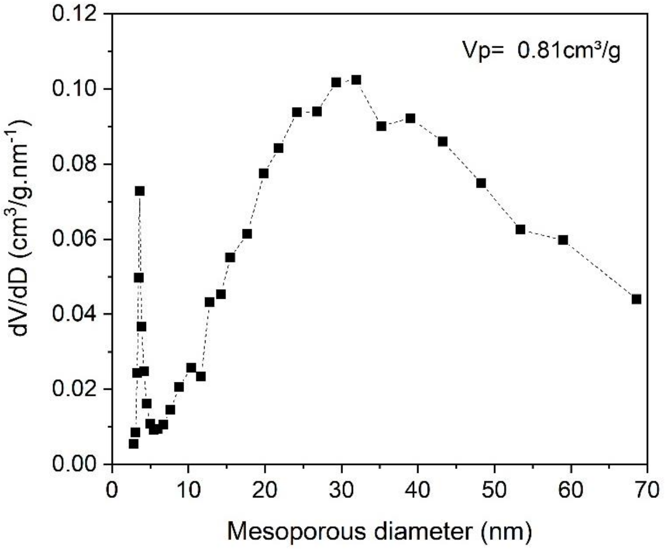

Figure S7: Pore size distribution of NiAl@Alg-Ca beads

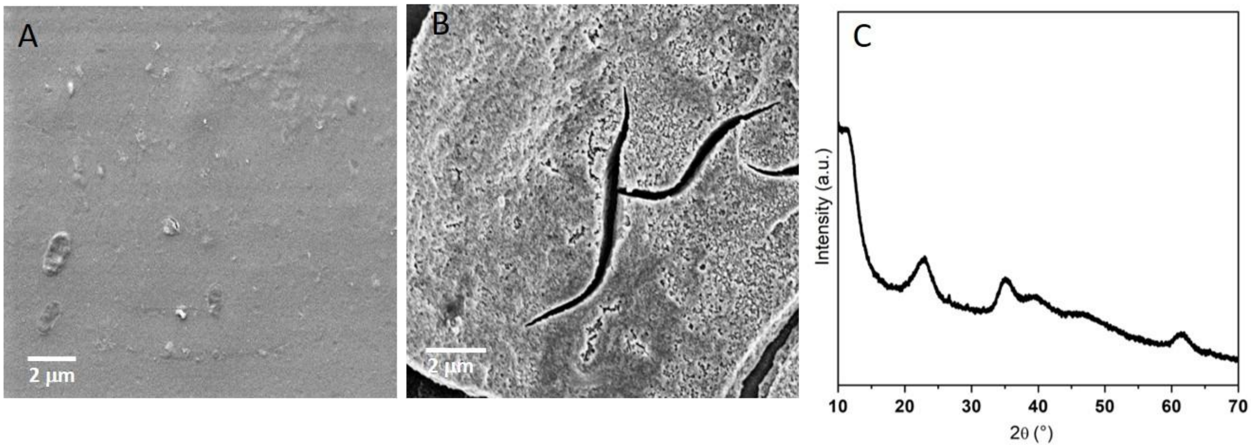

Figure S8: SEM images of A) ITO substrate coated with alginate thin film, B) ITO substrate coated with a NiAl@Alg-Ca film , C) PXRD patterns of NiAl@Alg-Ca film.

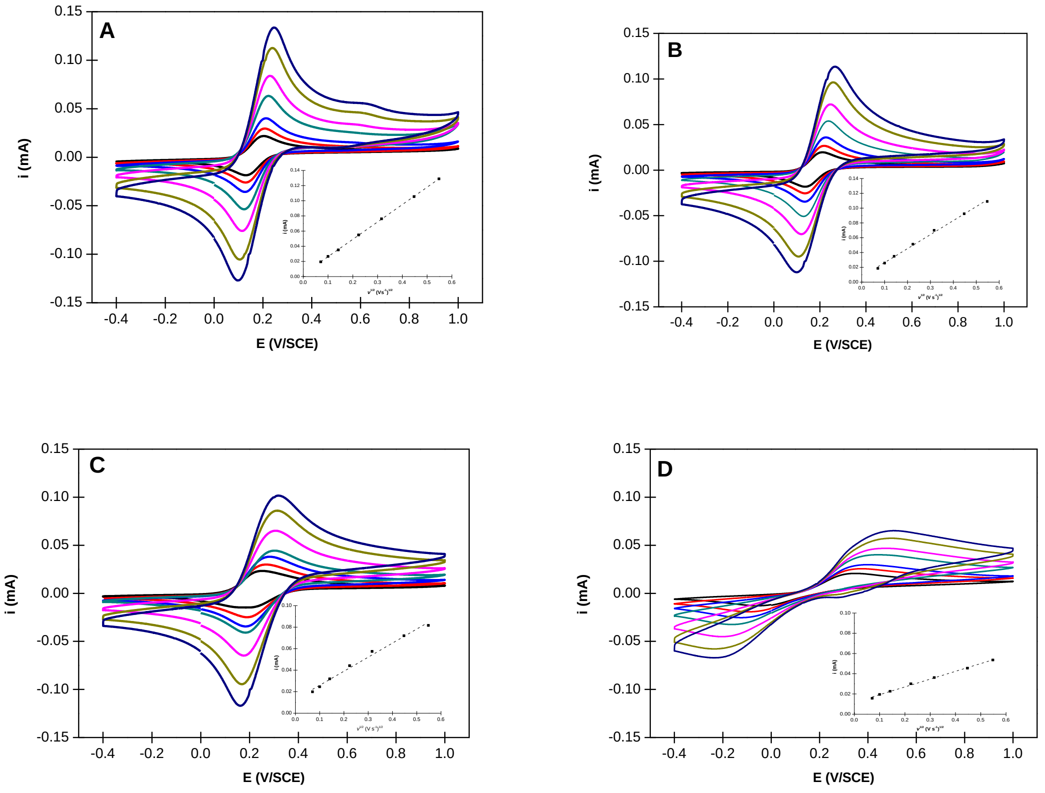

Figure S9: Cyclic voltammograms of 1 mM $K_4Fe(CN)_6$ in 0.1 M Tris buffer (pH 7) as a function of the scan rate (5-300 mV s^{-1}) recorded at A) NiAl@Alg-Ca/ITO and B) NiAl@Alg/ITO, C) NiAl-Alg/ITO and D) NiAl-CO_3/ITO.

Table S1 : Mass loss processes of the sodium alginate and ALg-Ca beads dried in the stove at 40°c and under CO_2 SC conditions

Sample	Attribution	ΔT	Δm (%)	Total Δm (%)
Na alginate	dehydratation	23.7 – 186.4	16.4	93.5
	decomposition	186.4 – 559.2	46.8	
	Na_2CO_3 formation	559.2 – 814.6	16.1	
	Na_2CO_3 decomposition	814.6 – 1102.2	14.2	
Alg-Ca $_{STOVE}$	dehydratation	24.6 – 180.1	14.7	88.1
	decomposition	180.1 – 401.0	42.0	
	$CaCO_3$ formation	401.0 – 590.2	22.9	
	$CaCO_3$ décomposition	590.2 – 695.0	8.5	
Alg-Ca $_{CO2\ SC}$	dehydratation	21.8 -130.1	44.3	91.0
	Alg decomposition	130.1 -403.4	29.6	
	$CaCO_3$ formation	403.4 – 580.3	11.5	
	$CaCO_3$ decomposition	580.3 – 699.3	5.6	
